# Vitamin D Levels in the Pre- and Post-COVID-19 Pandemic Periods and Related Confinement at Pediatric Age

**DOI:** 10.3390/nu15092089

**Published:** 2023-04-26

**Authors:** Caterina Mosca, Angelo Colucci, Fabio Savoia, Camilla Calì, Margherita Del Bene, Giusy Ranucci, Antonio Maglione, Angela Pepe, Annalisa Morelli, Pietro Vajro, Claudia Mandato

**Affiliations:** 1 Department of Translational Medical Science, Section of Pediatrics, University Federico II, 80131 Naples, Italy; 2 Department of Medicine, Surgery and Dentistry “Scuola Medica Salernitana”, Pediatrics Section, University of Salerno, 84081 Baronissi, Italy; 3 Epidemiology, Biostatistic and Childhood Cancer Registry of Campania, Santobono-Pausilipon Children’s Hospital, 80129 Naples, Italy; 4 Pediatric Department for the Treatment and Study of Abdominal Diseases and Abdominal Transplantation, IRCCS ISMETT (Mediterranean Institute for Transplantation and Advanced Specialized Therapies), University of Pittsburgh Medical Center, 90127 Palermo, Italy; 5 Pediatric Emergency Unit, Santobono-Pausilipon Children’s Hospital, 80129 Naples, Italy

**Keywords:** vitamin D deficiency, children, chronic diseases, COVID, lockdown

## Abstract

Coronavirus disease 2019 (COVID-19) restrictions have been correlated with vitamin D deficiency in children, but some uncertainties remain. We retrospectively studied vitamin 25-(OH) D blood levels in 2182 Italian children/adolescents hospitalized for various chronic diseases in the year before (n = 1052) and after (n = 1130) the nationwide lockdown. The type of underlying disease, gender, and mean age (91 ± 55 and 91 ± 61 months, respectively) of patients included in the two periods were comparable. Although mean levels were the same (*p* = 0.24), deficiency status affected a significantly higher number of subjects during the lockdown period than in the pre-COVID period (*p* = 0.03), particularly in summer (*p* = 0.02), and there was also a smoothing of seasonal variations in vitamin D levels. Particularly at risk were males (OR = 1.22; *p* = 0.03), the 1–5 year age group (OR = 1.57; *p* < 0.01) and the 6–12 year age group (OR = 1.30; *p* = 0.04). Infants appeared not to be affected (*p* = 1.00). In the post-COVID period, the risk of vitamin D deficiency was unchanged in disease-specific groups. However, the proportion of *deficiency* or *severe deficiency* differed significantly in the subgroup with endocrinopathy (higher; Chi-square *p* = 0.04), and with respiratory problems and obesity (lower; Chi-square *p* = 0.01 and *p* < 0.01, respectively). Conflicting/opposite literature results advocate for further studies to clearly indicate the need for supplementation during possible future periods of confinement.

## 1. Introduction

An optimal serum vitamin D status is fundamental for the promotion of health both at the pediatric age and in adulthood [1]. Beyond regulating the calcium-phosphate metabolism, vitamin D has been found to have a vital role at all ages concerning bone mass and metabolism, and also multiple central extra-skeletal effects on several target organs, such as adipose tissue, blood cells, the immune system, skin, muscles, endocrine pancreas, and blood vessels. Recent years’ relentless interest raising in vitamin D has been made possible and fueled by the discovery of the molecular mechanisms by which this molecule acts as a hormone. Its receptor (VDR) mediating the hormonal function in several physiological processes is expressed in almost all organs and acts via the genomic associated (nuclear VDR) and the non-genomic pathways (membrane VDR). The human body’s vitamin D is generated in large part through the skin synthesis of the provitamin dehydrocholesterol under the influence of ultraviolet radiation. Diet is a secondary minor source of cholecalciferol/vitamin D3 and ergocalciferol/vitamin D2 forms of vitamin D in animal and in plant products, respectively. Vitamin D so obtained is biologically inert and must undergo two hydroxylation reactions in the body to become active. The first occurs in the liver and converts vitamin D to 25-hydroxyvitamin D [25(OH)D], also known as “calcidiol”; the second happens mostly in the kidney and produces the physiologically active 1,25-dihydroxyvitamin D [1,25(OH)2D], also known as “calcitriol” [2,3].

Having said that, it appears clear that factors that may influence vitamin D status therefore include sun exposure, skin pigmentation/race, season, BMI, and nutritional factors (consumption of vitamin D-rich foods or vitamin supplements). In recent decades, adolescents have seemed to be particularly at risk for hypovitaminosis D, due to an increased tendency to conduct a sedentary indoor life with an increased access to computers, televisions and smartphones [4,5]. More recently, the coronavirus disease 2019 (COVID-19) pandemic determined the further confinement of adults and children for a very long period, with possible significant short-term and long-term effects on health. Confinement definitely reduced exposure time to sunlight, especially for people who do not live in rural areas, determining decreased cholecalciferol synthesis in the lower layers of the epidermis, which is the major source of vitamin D at all ages. Epidemiological studies have reported that the COVID-19 pandemic changed vitamin D levels in school and pre-school children [6,7,8]. Patients suffering from chronic diseases such as obesity, intestinal or hepatobiliary/pancreatic pathologies, pharmacologically treated epilepsy, and cancer have been described as being at an even higher risk of vitamin D deficiency [9,10], which could have been worsened during pandemics, but data from the COVID-19 period are lacking or conflicting. Our study aimed to fill these existing gaps and analyze the effects of the COVID-19 global-pandemic-related lockdown on the serum levels and status of 25-(OH)D in a cohort of children seen at a single pediatric third level hospital. We evaluated subgroups of children affected by several types of chronic diseases, also considering age, gender, season.

## 2. Materials and Methods

On 21 February 2020 the first case of COVID-19 was diagnosed in Italy, and a few weeks later, on 11 March 2020, the WHO officially declared it a pandemic. The Italian government officially announced a nationwide lockdown on 9 March 2020; this decree implied the total closure of schools, universities, public squares and all shops except supermarkets, grocery stores and pharmacies, as well as clearly defined travel restrictions [11]. We retrospectively enrolled children aged 1 month to 17 years of age that had performed a routine measurement of serum vitamin D levels during a hospitalization or a day in hospital in any ward of the third level Pediatric Hospital Santobono–Pausilipon in Naples between 11 March 2019 and 11 March 2021. Enrolled subjects were subsequently assigned to one of two groups, depending on when they had been hospitalized: the “pre-COVID group” for children hospitalized between March 2019 and March 2020, and the “post-COVID group” for children hospitalized between March 2020 and March 2021.

For the purpose of statistical analysis, each group was then split into further subgroups based on age range (0–12 months, 1–5 years, 6–12 years, >12 years; Table S1), gender (male, female; Table S2) or underlying pathology (endocrine, short stature, hepatic, nephrological, hematological, pneumological, obesity, neurological, oncological, gastroenterological, or other; Table S3). To evaluate the effects of the COVID-19-related lockdown on the serum concentration of vitamin D during different seasons, we performed separate analyses for each season, thus comparing pre-pandemic mean values to post-pandemic ones in the same season of the year. Serum samples were stored at 2–8 °C before assay.

Due to its quite long circulating half-life, serum concentrations of 25(OH)D rather than those of 1,25(OH)2D are the main acknowledged indicator of vitamin D status, reflecting vitamin D produced endogenously and vitamin D obtained from foods and supplements [2]. We measured the 25(OH) D concentration using automated electrochemiluminescence assay (ECLIA). For patients who had received more than one measurement of vitamin D, only the first collected value was considered for the analysis. Following the most recent recommendations released by international organizations [12] and Italian pediatric scientific societies [13], the serum vitamin D status of each study subject was evaluated in good agreement with those of the expert committee of the Food and Nutrition Board (FNB) at the National Academies of Sciences, Engineering, and Medicine (NASEM) [2]. In fact, 25(OH)D serum levels of 30 ng/mL (75 nmol/L) or greater were deemed *sufficient*, levels between 20 ng/mL (50 nmol/L) and 29 ng/mL were considered *insufficient*, subjects with serum 25(OH)D values below 20 ng/mL were considered as having vitamin *deficiency*, and levels below 10 ng/mL were classified as *severe vitamin D deficiency*.

Statistical analysis was performed using STATA software version 17 (StataCorp LP, College Station, TX, USA). Data are presented as absolute numbers, percentages, mean values and standard deviations. Taking into account that the obtained data did not display a normal distribution in our study population, and that there was a significant difference in the size of the groups, the non-parametric Kruskal–Wallis test was used to ensure the equality of the mean values amongst the groups. A Chi-square test was used to compare percentages amongst the different groups. The risk estimation between vitamin D status and the pre- and post-pandemic periods for the main potentially associated factors was analyzed. The response variable was dichotomized (vitamin D *deficiency* vs. *non-deficiency*) and the odds ratios with their corresponding *p*-value were calculated. The serum vitamin D level is asymmetrical and not normally distributed. In order to assess the effect on vitamin D levels of gender, age groups, seasons, and period (pre-COVID and post-COVID), generalized linear models (GLM) were applied with gamma distribution and inverse link function [14]. Furthermore, the presence of any interactions between the pandemic period and the other covariates was evaluated. A *p*-value level < 0.05 was considered statistically significant.

## 3. Results

Over the period under investigation, 2182 patients underwent a first serum vitamin D level measurement. Of them, 1052 patients had been hospitalized in the “pre-COVID” period (males = 549; females = 503; mean age 91 ± 55.08 months), while 1130 subjects had been hospitalized in the “post-COVID” period (males = 588; females = 542; mean age 91 ± 61.95 months). There were no statistically significant differences in age (*p* = 0.83) and gender (*p* = 0.94) distribution between the two groups.

**Figure 1:**
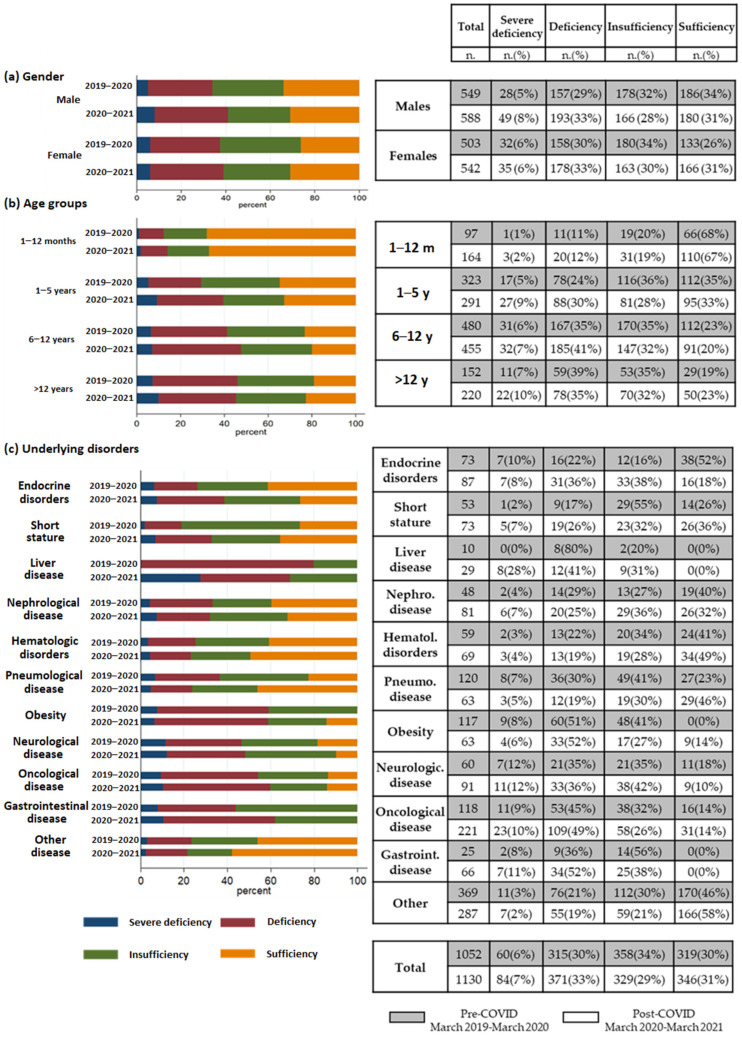
Comparison of serum vitamin D status by gender (**a**), age groups (**b**) and underlying disorders (**c**) in the pre- and post-COVID period (absolute frequencies, percentages).

Mean levels of vitamin D in the pre- and post-COVID groups (26.37 ng/mL vs. 25.67 ng/mL; *p* = 0.24) were comparable; however, there were more subjects with vitamin D *severe deficiency* or *deficiency* in the post-COVID group compared to the pre-COVID group (*severe deficiency* or *deficiency*: 40% vs. 36%; OR = 1.38, *p* = 0.01). (Figure 1) When studying different age groups, the 1–5 years and 6–12 years subgroups showed a significantly larger proportion of subjects with severely deficient or deficient vitamin D in the post-COVID period, with a higher risk of vitamin D *deficiency* (1–5 years: *severe deficiency* or *deficiency*: 39% vs. 29%; OR = 1.57, *p* < 0.01; 6–12 years: *severe deficiency* or *deficiency*: 48% vs. 41%; OR = 1.30, *p* = 0.04). (Figure 1) (Table S1) On the other hand, infants did not show abnormal values in either period. By analyzing the two genders separately, males had a significantly higher level of vitamin D *severe deficiency* or *deficiency* in the post-COVID group compared to the pre-COVID group (*severe deficiency or deficiency*: 41% vs. 34%; OR = 1.22, *p* = 0.03) (Figure 1) (Table S2).

Regarding the underlying diseases of our study population in the two periods, they were characterized by 11 subgroups: endocrinopathies (n = 73 and 87); short stature (n = 53 and 73); hepatopathy (n = 10 and 29); nephropathy (n = 48 and 81); hematological disorders (n = 59 and 69); pulmonary disease (n = 120 and 63); obesity (n = 117 and 63); neurological disorders (n = 60 and 91); oncological disease (n = 118 and 221); gastroenterological disorders (n = 25 and 66); and other diseases (n = 369 and 287). The vitamin D status of each of these subgroups is shown in Figure 1 and detailed in Table S3. The statistical analysis of the subgroups based on the underlying disorders of the studied subjects showed a relationship between vitamin D status and period, with higher percentages of vitamin D *deficiency* or *severe deficiency* in individuals affected by endocrine disorders (*severe deficiency*: 8% vs. 6%; *deficiency*: 31% vs. 20%; Chi-squared *p* = 0.04). (Figure 1). Furthermore, a significant association was observed between vitamin D status and pandemic period, with a lower and similar proportion of *deficiency* and *severe deficiency* in the post-COVID period, respectively, for patients with respiratory problems and obesity (Chi-squared *p* = 0.01 and *p* < 0.01, respectively). However, by calculating the odds ratios, no disease-specific group showed a statistically significant change in the risk of vitamin D deficiency during the post-pandemic period.

**Figure 2:**
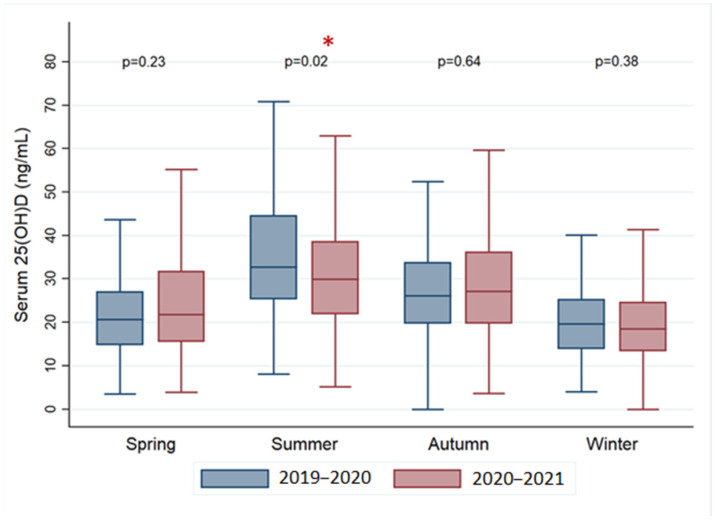
Comparison of the mean concentrations of serum vitamin D during the different seasons in the pre- and post-COVID period. * statistically significant.

Lastly, by comparing values obtained over the course of different seasons, only during the summer season were there significantly lower concentrations of serum vitamin D in the post-pandemic group compared to the previous period (*p* = 0.02). This was accompanied by a perceptible smoothing of the vitamin D levels peak of the summer seasonal variability versus the other periods in the same year (Figure 2).

In a multivariate analysis, we assessed the effect of gender, age group, season, and period on vitamin D levels (Table 1).

After adjusting for the covariates, the impact of the pandemic period on vitamin D levels is evident for gender (*p* < 0.05) and age (*p* < 0.01), with the first year of life showing significantly higher levels (mean 39.28 mg/dL). A significant interaction between seasonality and the difference between the pre- and post-pandemic periods (*p* < 0.01) emerges, as well.

## 4. Discussion

The importance of vitamin D in maintaining overall health cannot be ignored. It plays, in fact, a central role, especially in calcium absorption, bone mineralization, and immune system function, so that its deficiency is deemed responsible for a range of health issues which include, among others, rickets, osteoporosis, and increased susceptibility to autoimmune diseases and infections. The COVID-19 pandemic-related lockdown therefore brought some attention to the impact of confinement on the health of children during this exceptional period. Our retrospective, monocentric investigation was aimed at examining the correlation between the Italian nationwide lockdown measures implemented during the COVID-19 pandemic and the risk of vitamin D *deficiency* in a pediatric population. The study draws on a large cohort, therefore providing robust evidence to support the findings that the incidence of hypovitaminosis D during the pandemic was, in general, higher than in the pre-COVID period, with a greater prevalence among males and children aged 1–5 and 6–12 years. The above differences were particularly evident during the summer season, suggesting that these age groups may have been most affected due to the confinement-related reduced opportunities for outdoor activities and exposure to sunlight.

Seasonal fluctuations in serum vitamin D usually demonstrate the lowest values in winter and autumn and the highest values in summer and spring. Although in most geographic regions seasonal changes by themselves do not cause significant decreases in serum vitamin D, they can, however, result in lower levels in individuals at risk of vitamin D deficiency. In all cases, it has also been shown that the seasonal difference can be corrected to some degree with the help of vitamin D supplementation [15]. In this regard, it is worth noting that the usual routine supplementation of vitamin D for Italian infants under 12 months suggested by the scientific pediatric societies [13,16] may have therefore played a likely protective role, explaining the lack of significant differences in this age group between the pre- and post-COVID periods.

Previous surveys in the pediatric population have already reported a correlation between the global pandemic and vitamin D deficiency. A recent meta-analysis of a cohort of approximately 2000 individuals per period reported in five studies (of which four were retrospective and two focused specifically on infants) showed a significant decrease in vitamin D levels during the pandemic in all pediatric age groups except infants, as compared to the pre-pandemic period [17]. However, when the meta-analysis considered different classes based on their vitamin D status, the proportions of cases with vitamin *deficiency* or *insufficiency* remained unchanged [17], whereas we could register a *severe deficiency* of vitamin D only in the 1–5 and 6–12 year subgroups. Some differences among studies (e.g., smoothing of the vitamin D levels’ seasonal variability only in some cohorts) [18] may be attributable to the geographical heterogeneity of the studies included in the meta-analysis (Greece, Poland, Hong Kong, Korea, China), national differences in lockdown severity/duration, and vitamin D prescription habits by pediatricians, as well. Other factors which may have influenced vitamin D levels during the lockdown-related closure of schools likely comprise sociocultural and dietary aspects, including the consumption of processed and/or highly caloric foods and an excessive intake of sodas [19]. House confinement during the COVID-19 pandemic led, in fact, to dramatic shifts in the usual household food dynamics, likely impacting the health of all the family members. In this regard, a recent systematic literature review of parents’ perspectives on the changes in meal preparation and eating routines and other nutrition-related behaviors has shown that, in general, families were eating more varied foods and balanced home-cooked meals. On the other hand, however, these virtuous changes were combined with overeating and increased snacking on high-calorie snacks, desserts, and sweets. Moreover, food insecurity increased among the lowest-income families [20]. Altogether, all these elements are in agreement with the increased incidence of overweight, obesity and type 2 diabetes mellitus detected during the lockdown confinement [21].

When our results were broken down by the category of patients’ underlying disease, a surprising lower prevalence of vitamin D *deficiency* in children with obesity or respiratory problems emerged. Traditionally, obesity has been shown to have a negative effect on vitamin D levels due to a reduced bioavailability as a result of its adiponectin-mediated deposition in the adipose tissue compartments at all ages. Our study, instead, found a lower percentage of vitamin D *deficiency* in this group of patients, contrary to what had been previously observed by others [22,23,24]. However, when interpreting these findings, one should consider the likely possibility of a self-prescribed or medically recommended vitamin D supplementation. In fact, because individuals with obesity and chronic respiratory disorders, particularly atopic ones [13], were recognized to be at higher risk for more severe COVID-19 infections, vitamin D supplementation has become a widely practiced measure among these two categories of patients [25,26,27,28].

### Limitations of the Study

Despite the large sample size, representing a major strength of our study, there are some potential limitations that should be acknowledged. One possible drawback that we have already mentioned is the lack of comprehensive patient clinical information, including regarding their possible vitamin D supplementation. Additionally, the generalizability of our findings may be limited to populations with a similar sun exposure index and food/nutraceutical intake. Furthermore, the observational nature of the study, while demonstrates associations, does not allow the drawing of definitive conclusions about causality. Careful consideration of these limitations is therefore necessary when interpreting the results of our study, and further research is needed to confirm and extend our findings.

## 5. Conclusions

In conclusion, the global pandemic and its associated lockdowns have had a significant impact on the health of children, and one area of concern is the effect on serum vitamin D levels in this population. Our research confirms that the lockdown did indeed affect these levels, with a higher incidence of hypovitaminosis D in schoolers and pre-schoolers. Interestingly, infants appeared to be less affected, with normal values being maintained, likely due to the vitamin D supplementation recommended in this age group regardless of whether they are breastfed or formula-fed [13]. Similarly, the unexpected [29] higher percentage of vitamin D *sufficiency* observed in children with obesity and respiratory disorders may be due to the therapeutic or preventive administration of vitamins in these patients groups, as suggested by Italian primary care pediatric recommendations [13]. Much is probably also due to the wave of recent literature reports regarding these categories’ risks during the pandemic [26,27,28]. These issues should be considered in the contingency of future lockdowns.

The findings from our research add to the growing body of evidence regarding the vitamin D status of schoolers and pre-schoolers during periods of lockdown. This highlights the need for pediatricians to be vigilant about monitoring vitamin D levels in their patients, and to prescribe supplementation when necessary. Policymakers should consider the potential health consequences of future prolonged pediatric age individuals’ confinement, including their exposure to sunlight and opportunities for outdoor activities. Obesity prevention and treatment initiatives, as well as ongoing efforts to address nutritional management, parental feeding practices, and food insecurity, may account for these changes in the future [20]. Moreover, since optimal serum levels of vitamin D for bone and general health may not be the same and are also likely to fluctuate with life stages, race, ethnicity, and the specific measures used for assessing a wide variety of physiological processes, further studies appear necessary to determine which are the optimal indications for vitamin D supplementation [2,3].

## Figures and Tables

**Table 1:** Multiple generalized linear model.

			Vitamin D Level (ng/mL)	*p*-Value
Gender		**n**	**Mean (Standard Deviation)**	
	Males	1137	26.44 (14.78)	<0.05
	Females	1045	25.55 (13.26)	
Age				
	1–12 months	261	39.28 (18.05)	<0.01
	1–5 years	614	27.14 (14.73)	
	6–12 years	935	22.99 (10.80)	
	>12 years	372	22.45 (11.01)	
Season				
	Spring	521	24.30 (14.84)	0.18
	Summer	466	33.48 (14.15)	
	Autumn	466	28.12 (13.49)	
	Winter	592	20.89 (10.77)	
Period				
	pre-COVID	1052	26.38 (14.13)	0.60
	Post-COVID	1130	25.67 (14.00)	
Period * Gender				0.06
Period * Age				0.29
Period * Season				<0.01

* Interactions between the pandemic period and the other covariates.

## Data Availability

The data presented in this study are available on reasonable request from the corresponding author.

## Supplementary Materials

The following supporting information can be downloaded at: https://www.mdpi.com/article/10.3390/nu15092089/s1, Table S1: Comparison of serum vitamin D status in different age groups in the pre- and post-pandemic period; Table S2: Comparison of the serum vitamin D levels between males and females in the pre-COVID and post-COVID periods; Table S3: Comparison of vitamin D serum levels/status based on underlying disorders in the pre- and post-COVID era.
