# Supplementary material for: Vitamin D Levels in the Pre- and Post-COVID-19 Pandemic Periods and Related Confinement at Pediatric Age
Source: Nutrients. 2023 Apr 26;15(9):2089. doi: 10.3390/nu15092089 (PMC10181113; doi:10.3390/nu15092089)
Supplement: Supplementary file 1 [file nutrients-15-02089-s001.zip › nutrients-2306159-supplementary.pdf]

SUPPLEMENTARY TABLES

Table S1. Comparison of serum vitamin D status in different age groups in the pre- and post-pandemic period.

	Pre-COVID (March 2019- March 2020)					Post-COVID (March 2020- March 2021)				
	**Total n.**	**Severely deficient n.(%)**	**Deficient n.(%)**	**Insufficient n.(%)**	**Sufficient n.(%)**	**n.**	**Severely deficient n.(%)**	**Deficient n.(%)**	**Insufficient n.(%)**	**Sufficient n.(%)**
**Age**										
1-12 m	97	1(1%)	11(11%)	19(20%)	66(68%)	164	3(2%)	20(12%)	31(19%)	110(67%)
1-5 y	323	17(5%)	78(24%)	116(36%)	112(35%)	291	27(9%)	88(30%)	81(28%)	95(33%)
6-12 y	480	31(6%)	167(35%)	170(35%)	112(23%)	455	32(7%)	185(41%)	147(32%)	91(20%)
>12 y	152	11(7%)	59(39%)	53(35%)	29(19%)	220	22(10%)	78(35%)	70(32%)	50(23%)
TOT	1052	60 (6%)	315 (30%)	358 (34%)	319 (30%)	1130	84 (7%)	371 (33%)	329 (29%)	346 (31%)

m, months y, years

Differences were all not statistically significant except for the number of Total subjects with vitamin D Deficient and Severely deficient status in the post-COVID group compared to the pre-COVID group (N= 455 vs 375; p=0.03).

Table S2 Comparison of the serum Vitamin D status between males and females in the pre-COVID and post-COVID periods.

	Pre-COVID (March2019- March 2020)					Post-COVID (March 2020-March 2021)				
	**Total n.**	**Severely deficient n.(%)**	**Deficient n.(%)**	**Insufficient n.(%)**	**Sufficient n.(%)**	**Total n.**	**Severely deficient n.(%)**	**Deficient n.(%)**	**Insufficient n.(%)**	**Sufficient n.(%)**
Males	549	28 (5%)	157 (29%)	178 (32%)	186 (34%)	588	49 (8%)	193 (33%)	166 (28%)	180 (31%)
Females	503	32 (6%)	158 (30%)	180 (34%)	133 (26%)	542	35 (6%)	178 (33%)	163 (30%)	166 (31%)

There were no statistically significant differences in gender (p = 0.94) distribution between the two periods.

Table S3. Comparison of Vitamin D status based on underlying disorders in the pre- and post-pandemic period.

	Pre-COVID (March 2019- March 2020)					Post-COVID (March 2020- March 2021)				
	**Total n.**	**Severely deficient n.(%)**	**Deficient n.(%)**	**Insufficient n.(%)**	**Sufficient n.(%)**	**Total n.**	**Severely deficient n.(%)**	**Deficient n.(%)**	**Insufficient n.(%)**	**Sufficient n.(%)**
**DIAGNOSIS**										
Endocrinopathy	126	8(6%)	25(20%)	41(33%)	52(41%)	160	12(8%)	50(31%)	56(35%)	42(26%)
Short Stature	53	1(2%)	9(17%)	29(55%)	14(26%)	73	5(7%)	19(26%)	23(32%)	26(36%)
Hepatopathy	10	0(0%)	8(80%)	2(20%)	0(0%)	29	8(28%)	12(41%)	9(31%)	0(0%)
Nephropathy	48	2(4%)	14(29%)	13(27%)	19(40%)	81	6(7%)	20(25%)	29(36%)	26(32%)
Haematological disorder	59	2(3%)	13(22%)	20(34%)	24(41%)	69	3(4%)	13(19%)	19(28%)	34(49%)
Pulmonary disease	120	8(7%)	36(30%)	49(41%)	27(23%)	63	3(5%)	12(19%)	19(30%)	29(46%)
Obesity	117	9(8%)	60(51%)	48(41%)	0(0%)	63	4(6%)	33(52%)	17(27%)	9(14%)
Neurological disorder	60	7(12%)	21(35%)	21(35%)	11(18%)	91	11(12%)	33(36%)	38(42%)	9(10%)
Oncological disease	118	11(9%)	53(45%)	38(32%)	16(14%)	221	23(10%)	109(49%)	58(26%)	31(14%)
Gastroenterological disorders	25	2(8%)	9(36%)	14(56%)	0(0%)	66	7(11%)	34(52%)	25(38%)	0(0%)
Other	369	11(3%)	76(21%)	112(30%)	170(46%)	287	7(2%)	55(19%)	59(21%)	166(58%)
Total	1052	60(6%)	315(30%)	358(34%)	319(30%)	1130	84(7%)	371(33%)	329(29%)	346(31%)

Differences were all not statistically significant except for the subgroup with endocrinopathy (higher risk of *deficiency* or *severe deficiency* in the post-pandemic period; p=0.04), and for the subgroup with respiratory problems and with obesity (lower risk of *deficiency* or *severe deficiency* in the post-pandemic period; p=0.01 and p<0.01, respectively).
